# Impact of the Addition of *Tenebrio molitor* and *Hermetia illucens* on the Physicochemical and Sensory Quality of Cooked Meat Products

**DOI:** 10.3390/insects14050487

**Published:** 2023-05-22

**Authors:** Barbara Lemke, Lisa Siekmann, Nils Th. Grabowski, Madeleine Plötz, Carsten Krischek

**Affiliations:** Institute of Food Quality and Food Safety, University of Veterinary Medicine Hannover, Foundation, Bischofsholer Damm 15, 30173 Hannover, Germany

**Keywords:** *Tenebrio molitor*, *Hermetia illucens*, cooked meat product, physicochemical parameters, sensory analysis, microbiological quality

## Abstract

**Simple Summary:**

The growing need for protein cannot be covered in the future by traditional farming of cattle or pigs, especially considering its problematic impact on climatic greenhouse gas emissions. One alternative protein source is insects, which have less-negative impacts on these emissions. However, in many (European) countries, the consumption of insects is less accepted by consumers. An alternative might be the use of insects in meat products, which are popular among consumers. The problem is that consumers have expectations about these products that should be met when insects are added to the meat products. Therefore, in this study, we produced a cooked meat product and replaced 10% and 20% of the meat with whole insect larvae powder from yellow mealworms and black soldier flies. This replacement negatively influenced the liquid losses during cooking, the color and protein results, and after storage in modified atmosphere packages, the color, sensory and hardness results of the meat products, especially after replacing 20% of the meat with insects and after adding black soldier fly larvae. The growth of bacteria added to the surface of the products during storage was not altered by the insect replacement.

**Abstract:**

The use of proteins from insects, plants, microalgae, fungi or bacteria as an alternative to proteins of animal origin such as meat, fish, eggs or milk can meet the worldwide protein demand in the future. As the consumption of whole insects might be problematic or unacceptable for many consumers, especially in European countries, the use of homogenized insects or protein extracts from insects for the production of products might be a possibility to overcome general acceptability problems. However, the quality criteria of these products have to be comparable with consumers’ expectations with regard to known products. Therefore, in the present study, we produced a meat product, replaced 10% and 20% of the pork with homogenized larvae of *Tenebrio molitor* and *Hermetia illucens*, and determined different physicochemical and sensory parameters at production and during modified atmosphere storage for 21 days. Additionally, the alteration of different bacteria species during this storage was analyzed in challenge tests. After production, the addition of insects resulted in higher cooking losses and pH values in the products with 20% insects, higher pH and yellowness, lower lightness, protein and hardness results in the *Hermetia* products, as well as higher yellowness and lower protein and hardness values in the cooked meat products with *Tenebrio molitor*. During modified atmosphere storage, the color differences principally remained, whereas the concentrations of inoculated *Bacillus cereus*, *Listeria monocytogenes* and *Escherichia coli* were not influenced by the addition of insects to the cooked meat products. The sensory results of the insect products, especially at higher concentrations and with *Hermetia illucens*, worsened during modified atmosphere storage. The addition of homogenized insect larvae, especially at higher concentrations and particularly of *Hermetia illucens*, influences different physicochemical and sensory parameters of the cooked meat products.

## 1. Introduction

Meat products are a fundamental part of the diet in European countries [1]. Nevertheless, the consumption of these products might also be related to an increased risk of specific diseases (e.g., cancer and heart problems), especially if the products are consumed more than usual and/or contain specific (potentially problematic) ingredients (e.g., salt or nitrite) [2,3]. Besides these potential health problems, the production of poultry, beef, or pork is critically evaluated by certain consumer groups as it has a negative impact on the environment. This mainly includes the emission of carbon dioxide in relation to the production of feed (necessary for these animals) and carbon dioxide and methane in relation to the husbandry of the animals [1]. Considering the latter and as the protein demand continually increases, for example, due to the growth of the global population and higher prosperity in (former developing) countries, improvement of meat production efficiency can and should only partly meet the increasing worldwide protein demand. Therefore, other protein sources should be evaluated as an alternative to meat to ensure food supply security with a reduced impact on the environment [1].

Insects are an interesting alternative protein source for human consumption considering that rearing insects usually has a lower environmental impact compared to beef or pork. For example, frozen, dried, and powder forms of the yellow mealworm (*Tenebrio* (*T.*) *molitor* larva) were approved as a novel food for human consumption in the European Union in 2022. In the review by Wang and Shelomi [4] from 2017, the authors stated that the black soldier fly larvae? (*Hermetia* (*H.*) *illucens*) is consumed mainly by humans of rural or traditional cultures and that the consumption is neither popular nor common. However, there is an application for approval by the European Union for a *Hermetia* meal (based on the dried ground meal of *H. illucens* larvae) as a novel food [5], and *H. illucens* has been recently assessed by the EFSA with regard to allergenicity as a potential future source for alternative food proteins [6]. Considering this, the approval of *H. illucens* as a novel food in the European Union could be expected. Therefore, we used the latter insect species as well as *T. molitor* in the present study. The protein content of insect larvae from *T. molitor* or *H. illucens* is between 42 and 77% of the dry weight [7,8,9,10,11]. The protein content is influenced by factors such as the insect species and its instars, the diet during rearing, or the preparation of the insects before consumption. The protein contents of insects in relation to the dry weight in both insect species seem to be quite low in comparison to the protein contents of specific porcine muscles with up to 90% of the dry weight (*Musculus* (*M.*) *longissimus*, *M. semimembranosus*, recalculated to the dry weight [12,13,14]) or chicken muscles (*M. pectoralis*) with up to 96% of the dry weight [15,16], recalculated to the dry weight). However, the latter values are not directly comparable as in insects the protein content of the whole body is considered. The protein content of whole eviscerated pig carcasses is approximately 35 g protein/100 g dry weight [17] and, as the carcass yield is around 76% in pigs [18], the whole body protein content in pigs is only about 27% (considering that the dry weight of the organs and carcass is equal). Considering that in chicken the yield of the *M. pectoralis* without skin and small breast muscle is around 20% of the live weight [19], the protein content of the whole body is only 19% (considering that the dry weights of non-breast parts are equal). In conclusion, many insects have quite a good protein content compared to pork, beef, or chicken meat.

Fat is another important component of insects such as *T. molitor* or *H. illucens.* Analyses of *Tenebrio* and *Hermetia* show that the insects consist of 24.7 to 37% fat (on dry matter base) [7,8,9,10,11,20]. Therefore, insects are also a major fat source. However, fatty acid composition analyses indicate clear differences between *Tenebrio* and *Hermetia* with high concentrations of unsaturated fatty acids (UFA) of *T. molitor* [8,9] in comparison to *H. illucens* with higher contents of saturated fatty acid (SFA) contents [10,20]. With regard to the three most important fatty acids of both insect species, larvae of *Tenebrio* consist (in decreasing concentrations) of the UFAs oleic acid (C18:1) and linoleic acid (C18:2) and the SFA palmitic acid (C16:0) [8,9] and *H. illucens* larvae (in decreasing concentrations) of the SFAs lauric acid (12:0) and palmitic acid (C16:0) and the UFA oleic acid (C18:1) [10,20].

Another main component of insects is chitin, which is the second most abundant biopolymer on earth after cellulose and is found in crustaceans, fungi, and insects. For example, in insects such as *T. molitor* or *H. illucens*, chitin is found in concentrations of 50 to 150 g/kg dry matter [20,21,22,23]. Chitin is a hard, inelastic polysaccharide consisting of D-glucosamine and N-acetylated-D-glucosamine, linked by β-1,4 glycosidic bonds, and is insoluble in water and most organic solvents [24]. The possibility of chitin digestion by humans has long been questioned, but chitinase, the enzyme that degrades chitin, has been found in the gastric fluid of humans [25]; nevertheless, chitin could also be digested by bacteria and fungi within the colon of humans, making insects, if the chitin is not removed, a dietary fiber component of the food [24], which might influence the overall acceptability of products containing insects.

As the consumption of whole insects is not well accepted in many countries, it is an option to use homogenized insects or parts of them as well as extracted protein for the production of food products. This has been described, for example, for bread using *Acheta* (*A.*) *domesticus* (house cricket) and *T. molitor* [26], cooked sausages with the addition of *Zophobas* (*Z.*) *atratus* (superworms) [27], Frankfurter sausages using *T. molitor* [28], Vienna-style sausages with the addition of *H. illucens* larvae [29], and for meat batters added with *B. mori* (silk moth pupae) [30].

In many studies, dried or otherwise processed insects are used for the production of their products. These processes are time-consuming and less sustainable. Therefore, in the current study, whole larvae of *T. molitor* and *H. illucens* were used for the production of a cooked meat product by replacing 10% and 20% of the pork and beef with the insects. To imitate possible bacteria contamination during production, slicing, and packaging and changes in these contaminations during storage in retail, the meat products were subsequently inoculated with bacteria species found on insects and/or in meat processing units [31,32] and stored in modified-atmosphere packages for up to 21 days. The aim of the study was to elucidate whether the quality of the cooked meat products was influenced by the partial replacement of pork and beef by the insect homogenates directly after production and during modified atmosphere storage.

## 2. Materials and Methods

### 2.1. Experimental Design

In the study, within a two-week interval, three batches of living insect larvae of *T. molitor* or *H. illucens* were purchased from a commercial insect company and were killed by freezing in a −80 °C freezer. The latter procedure was chosen to ensure a quick and gentle killing of the insects on a laboratory scale, although we know that freezing at higher temperatures might be also appropriate for killing larger amounts of larvae on an industrial scale. The frozen insects were homogenized for 1 min at 10.000 rounds per min (rpm, Grindomix GM 200, Retsch GmbH, Haan, Germany). The homogenates were autoclaved to remove all contamination of microorganisms (including bacteria spores (e.g., of *Bacillus* spp.)), as in the present study whole and not dried or otherwise processed insects were used, which might be microbiologically contaminated despite the initial freezing procedure.

For the study, cooked meat products with five different compositions were produced (Table 1). The products were produced in three independent repeats; in each repeat, the same batch of insect larvae was used. All components were mixed and transferred to metal forms (length of 300 mm and height of 70 mm). The mixture was heated in an oven at 110–120 °C to a core temperature of 64 °C (controlled by inserting a digital thermometer) for 60 to 75 min. After cooling, the weights before and after heating/cooking were determined to calculate the percent of cooking loss. The color values were analyzed before and after cooking and cooling. Then, the pH values and the shear force values of the products were determined. Samples for analysis of the protein and fat content were collected, homogenized for 1 min at 10.000 rpm (Grindomix GM 200, Retsch), and stored at −20 °C until analysis. For all the repeats, a sufficient quantity of pork and beef (freed from visible fat) as well as pork fat were purchased in advance from a commercial slaughterhouse. These ingredients were cut, homogenized and frozen in plastic bags at −20 °C. One day before the day of production, the meat and fat were removed from the freezer and thawed in a chilling room. All meat products were produced within one month at two-week intervals to minimize the negative effects of frozen storage on the pork, beef, and pork fat.

For the storage experiments, round slices with an area of 25.5 cm^2^ were cut and inoculated with *Listeria* (*L.*) *monocytogenes*, *Escherichia* (*E.*) *coli*, and *Bacillus* (*B.*) *cereus*, respectively, by adding 100 µL of the bacteria suspension (ca. 5.0 × 10^6^ colony-forming units (cfu)) for *L. monocytogenes*/*E. coli*; ca. 1.0 × 10^4^ for *B. cereus*), prepared as described in Section 2.2 and quantified as described in Section 2.3, to the surface with even distribution of the suspension with an L-shaped spreader (VWR International, Darmstadt, Germany). The meat product samples were transferred to polypropylen trays (ES-Plastic GmbH, Hutthurm, Germany), packaged in 70% N_2_ and 30% CO_2_, and sealed with a polyethylen-ethylene vinyl alcohol-PP transparent film (Südpack, Ochsenhausen, Germany) in a packing machine (MultivacT100, Sepp Haggenmueller GmbH& Co. KG, Wolfertschwerden, Germany). The samples were stored at 7 °C until further analyses. The samples were examined microbiologically on days 0, 7, and 21 to elucidate the product-related effects on the bacteria at the beginning of retail storage or close to the end of the best-before date. Additionally, non-inoculated meat product samples were packaged and stored, as described before. On days 7 and 21, immediately after opening these packages, color and texture values and sensory properties and after homogenization water activity values (a_w_ values) of the products were determined. To ensure a comparable amount of bacteria on all meat products before packaging, the same suspension of the same bacteria species was added to the surface and the inoculated quantity was verified by the analysis of all products directly after packaging (day 0).

### 2.2. Preparation of the Microorganisms

For the storage experiments, *L. monocytogenes* DSM 20600, *E. coli* DSM 682 (German Collection of Microorganisms and Cell Cultures, DSMZ, Braunschweig, Germany) and *Bacillus cereus* (from a milk sample of the Institute of Food Quality and Food Safety) were used. All bacteria, previously stored in cryotubes in a −80 °C freezer, were transferred three to four days before the experiment to sheep blood agar (Oxoid GmbH, Wesel, Germany) and incubated at 30 °C. One day before the investigations, individual colonies from the sheep blood agar were transferred into brain heart infusion (BHI) broth and incubated for 24 h at 30 °C. This BHI bacteria suspension was used for the inoculation experiments. The bacteria number in the BHI bacteria suspension solution was always analyzed on the investigation day, as described in Section 2.3.

### 2.3. Microbiological Analyses

The inoculated meat product samples were weighed and transferred to bags (Stomacher 400 Strainer Bags, Seward Limited, Worthing, United Kingdom). After the addition of a ninefold sterile saline solution with peptone (0.85% NaCl and 0.1% peptone), the samples were homogenized for 2 min at 230 rpm with a stomacher (Stomacher 400 Circulator, Seward). After homogenization, the samples were serially diluted (1:10, up 10^6^). For analysis of the bacteria, 0.1 mL of each appropriate dilution was spread onto PEMBA agar (*Bacillus cereus* selective agar, for *B. cereus*, ISO 7932:2004), chromogenic coliform agar (*E. coli*, ISO 16649-2:2001) and OCLA (Oxoid chromogenic Listeria agar, for *L. monocytogenes,* ISO 11290-1:2017) (Oxoid) and incubated at 30 °C (*B. cereus*), 37 °C (*L. monocytogenes*) and 44 °C (*E. coli*) for 48 h.

The detection limits for all bacteria were 2.0 log_10_ cfu/g meat product. If no colonies were determined on the agar plates with the initial dilution, the half detection limit (1.7 log_10_ cfu/g meat product) was used for further calculations.

To analyze the total number of bacteria in the BHI suspension before inoculation, serial 10-fold dilutions of up to 10^7^ of the solution were performed using a saline solution with peptone (0.85% NaCl and 0.1% peptone). Measures of 0.1 mL of the 10^5^, 10^6^, and 10^7^ dilutions were pipetted on the appropriate agar plate and incubated, as presented above. The bacteria number was always at approximately 5.0 × 10^7^ cfu per mL for *E. coli* and *L. monocytogenes* and approximately 1.0 × 10^5^ cfu for *B. cereus*.

### 2.4. Physical and Chemical Analyses

The color of the meat product surface after cooking and on days 7 and 21 of modified atmosphere storage was determined with a Minolta CR 400 colorimeter (Konica-Minolta GmbH, Langenhagen, Germany; 2° standard observer, D65 illuminant, 8 mm measuring field). The apparatus was calibrated with a standard white plate (Konica-Minolta GmbH). The results are expressed as CIE L* (lightness), a* (redness) and b* (yellowness). During the storage experiments, color measurements were performed immediately after opening the packages. The color determination was performed in triplicates and the mean value of these repeats was used for further statistical analysis.

Texture profile analysis (TPA) was performed on days 7 and 21 of modified atmosphere storage of the meat product. The samples with a diameter of 2.2 cm and a thickness of 3.3 cm were analyzed using a texture analyzer (TA.XT.plus, Stable Micro Systems, Survey, United Kingdom) equipped with a 50 kg load cell and a round aluminum stamp (50 mm). The stamp moved down with a speed of 3 mm/s until 40% of the sample height was reached. The back speed was also 3 mm/s. The measurement was repeated once. The texture parameters hardness, gumminess, cohesiveness, and chewiness were calculated using the software. The TPA analysis was repeated five times and the mean value of these repeats was used for further statistical analysis.

The pH value was measured after cooking with a portable pH meter (Portamess^®^, Knick GmbH, Berlin, Germany) with a glass electrode (InLab 427, Mettler-Toledo, Urdorf, Switzerland) and a temperature sensor. Initially, the pH meter was calibrated with pH 4.0 and pH 7.0 standard solutions (Carl Roth GmbH + Co. KG, Karlsruhe, Germany). Each pH measurement was performed at three different points on the sample. The pH determination was repeated twice and the mean value of these repeats was used for further statistical analysis.

The protein concentration of the meat product after cooking was calculated by analysis of the nitrogen concentration, using the Kjeldahl method (Vapodest 50s^®^, Gerhardt Laboratory Systems GmbH, Koenigswinter, Germany) (ISO 937:1978, meat and meat products). The protein content was calculated using the nitrogen-to-protein factor 6.25 [9], as the main component of the meat product was pork and beef. Protein analysis was performed in triplicates and the mean value of these repeats was used for further statistical analysis.

The fat concentration of the meat product after cooking was determined after acid hydrolysis and extraction in Soxhlet equipment (LAT GmbH, Garbsen, Germany) (ISO 1443:1973). The fat analysis was performed in triplicates and the mean value of these repeats was used for further statistical analysis.

The water activity of the meat product was measured once with an a_w_-cryometer (AWK-40, NAGY Messsysteme GmbH, Gäufelden, Germany) on days 7 and 21 of modified atmosphere storage. For the calibration of the apparatus, a 10% NaCl solution was used.

### 2.5. Sensory Analysis

The sensory analysis was performed by three trained persons directly after opening the packages on days 7 and 21 of storage principally considering the sensory test conditions of the German Agricultural Society (DLG). The appearance and odor of the products were rated on a scale of 1 to 5 (5 = no deviation, 1 = unsatisfactory, unacceptable). For the calculation of the total sensory values, at first, the mean appearance and odor values of the three persons were calculated. As the appearance has a higher impact on the consumers’ buying decision, these results were multiplied by 3, whereas the odor results were not changed. The results of appearance and odor were then summed up. This value was divided by four to achieve a value between the sensory DLG scale of 1 to 5.

### 2.6. Statistical Analysis

The data of all experiments were statistically analyzed with SAS Enterprise Guide 7.1 (SAS Institute Inc., Cary, NC, USA) using the SAS PROC GLM procedure considering the following model:

Y_ijk_ = µ + S_i_ + C_j_ + SC_ij_+ R_k_ + ε_ijk_

where Y_ij_ = observation value; µ = overall mean; S_i_ = fixed effect of insect species or control; Cj= fixed effect of concentration (0%, 10%, 20%); SC_ij_ = fixed effect of interaction of S and C; R_k_ = random effect of repeat; and ε_ij_ = random error.

This was followed by the TUKEY multiple comparison test. All values were presented as means ± standard deviation (SD). Means were marked as significant if the *p*-value was lower than 0.05. All experiments were independently replicated at least three times.

## 3. Results and Discussion

### 3.1. Analysis of the Cooked Meat Product

**Figure 1:**
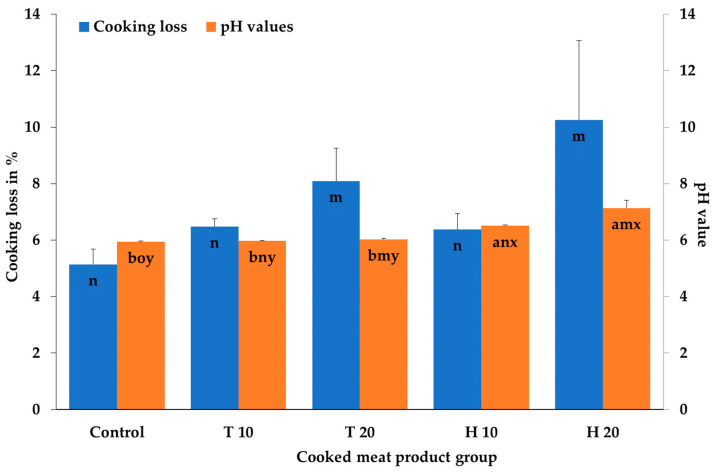
Mean and standard deviation values of the cooking losses and pH values after cooking of the cooked meat products depending on the meat product group; T 10/T 20 consist of 10%/20% homogenized *Tenebrio molitor* larvae, H 10/H 20 consist of 10%/20% homogenized *Hermetia illucens* larvae; ^ab^ bar graphs with different letters differ significantly (*p* ≤ 0.05) between the species (N = 6 per species) and controls (N = 3); ^mno^ bar graphs with different letters differ significantly (*p* ≤ 0.05) between the concentrations (0% (N = 3), 10% (N = 6), 20% (N = 6)); ^xy^ bar graphs with different letters differ significantly (*p* ≤ 0.05) between the interaction of species and concentrations (N = 3).

The products with 20% *H. illucens* or *T. molitor* showed significantly higher cooking losses than the control products and the cooked meat products with 10% of the insects. No significant effect of the insect species or the interaction of species and concentration could be obtained (Figure 1).

**Figure 2:**
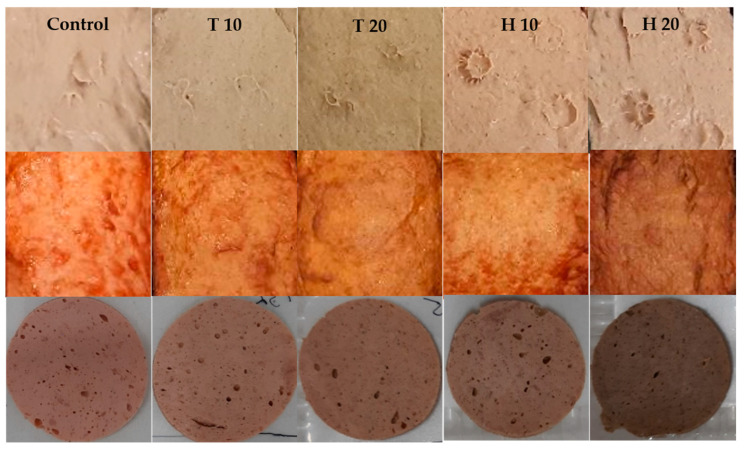
Images of the cooked meat products before (**top**) and after cooking (**center**) and of round slices of the products with an area of 25.5 cm^2^ before packaging in modified atmosphere packages (**bottom**) depending on the meat product group; T 10/T 20 consist of 10%/20% homogenized *Tenebrio molitor* larvae, H 10/H 20 consist of 10%/20% homogenized *Hermetia illucens* larvae.

The cooking loss during the heating of meat products such as sausages is a quality parameter that is frequently analyzed. Additionally, studies showing the effects of the addition of *Hermetia* to meat products on cooking losses have not been published until now. Experiments with cooked sausages [28] showed that the replacement of pork with more than 30% dried *T. molitor* also resulted in higher cooking losses. Kim et al. [33] found lower water-holding capacities in an emulsion system after the addition of *T. molitor*. Choi et al. [28] argue that the myofibrillar proteins in the insects were denatured during drying of the insects before their processing, which can influence the binding properties of these proteins. Kim et al. [33] explained the effect on the cooking loss with the reduction in myofibrillar protein, which usually has better efficiency in trapping water in insect products. In contrast to the present study, lower cooking losses were presented in cooked emulsion sausages after the addition of *T. molitor* larvae flour [34], in meat batters with *B. mori* pupae [30], after the addition of superworm (*Z. atratus*) larvae at higher concentrations to cooked sausages [27], or after the addition of *A. domesticus* to sausage patties [35]. However, in all of these studies, the products had higher protein concentrations, which might explain the differences. Besides the impact of the protein on the water binding, Park et al. [30] explained their lower cooking losses with the higher pH values. This is in accordance with Klettner [36] who showed that pH values are positively related to the water binding of cooked sausages. However, despite this, the relationship between the pH values and the cooking losses has been presented quite contradictorily. For example, Choi et al. [28] also found high pH values and high cooking losses. Ho et al. [35] found comparable pH values despite higher cooking losses in the insect products, whereas again Kim et al. [34] found high pH values and low cooking losses. The higher cooking losses in the present study might be explained by the reduction in and the denaturation of the myofibrillar proteins and the accompanied decrease in the water binding properties but not by the pH value. The addition of the insects in the present study reduces the protein content in the products (Figure 2) and the freezing and autoclaving of the insects and the cooking of the meat products might influence protein denaturation.

Considering the interaction effects of species and concentration, the *Hermetia* products with 10% and 20% had significantly higher pH values than the cooked meat products of all other groups. This result was also found with regard to the effect of the insect species and control. The products with *H. illucens* showed, independent of the concentration, significantly higher pH values than the control and *T. molitor* cooked meat products. The cooked meat products with 20% insects, independent of the species, had significantly higher pH values than the 10% and the control products, which also differed significantly (Figure 1).

In different studies that investigated cooked sausages or similar products after the addition of *T. molitor*, higher pH values were also determined [28,33,34]. However, the pH results of cooked products/sausages with *H. illucens* have not been published until now. Considering other insect species, Park et al. [30] and Kim et al. [34] also found higher pH values of emulsion sausages and cooked meat batters, respectively, after the addition of *B. mori*. It is interesting to note that the addition of *A. domesticus* to meat emulsions or cooked sausages did not result in an increase in the pH values [35,37]. Insects such as *T. molitor*, *H. illucens*, and *B. mori* have higher pH values of 6.4 to 7.4 [34,38] compared to pork with a final pH of 5.7 [12]. This might explain the higher pH values in the products with 20% insects and especially with *H. illucens*, as frozen larvae of *Hermetia* show pH values of up to 7.4 [38].

Considering the lightness results after cooking, the control cooked meat products had the highest L* values, differing significantly from the *Hermetia* products. The L* results of the products with *Tenebrio* were comparable with the values of the control and *Hermetia* cooked meat products. Products with 20% insects had significantly lower L* values than those with 10% insects.

The presented effect on the L* values of the cooked meat products was in agreement with other studies using *T. molitor* [28,33,34], *B. mori* [30,34], or grasshopper flour (*Sphenarium* (*S.*) *purpurascens*) [39]. However, the addition of *A. domesticus* [35] did not change the L* values. A reason for the darkening of the products might be the principally lower L* values, meaning a darker color of flours from insects such as *Tenebrio* [34] in comparison to pork [18].

With regard to the a* values after cooking the meat products, no effects of the addition of *Hermetia* and *Tenebrio* insects at different concentrations on these values could be found.

In studies that analyzed the a* values after heating/cooking, the addition of *T. molitor* also resulted, on the one hand, in comparable redness results [34], and on the other hand, in higher a* values at higher *Tenebrio* concentrations [28]. With other insects, lower a* values (*B. mori* [30,34], *A. domesticus* [35], *S. purpurascens* [39]) and similar a* values were found (*B. mori* [30]), *S. purpurascens* [39]). The vertebrate meat contains heme proteins such as myoglobin, which are responsible for the red color of the meat. This meat is partly replaced by insects, which do not consist of these proteins. After heating, processes such as denaturation or oxidation reduce the impact of these heme proteins on the color differences, which might explain the similar redness results after cooking.

After cooking, the b* values of the control cooked meat products were significantly lower compared to the results of the other groups, except for the products with 10% *T. molitor*. The latter had b* values after cooking comparable to those of all other groups. With regard to the effect of the species, cooked meat products with *T. molitor* and *H. illucens* had comparable b* values, independent of the concentration. However, the results were significantly lower than the control results (Table 2).

The b* results after cooking mainly correspond with other publications (*T. molitor* [28,33], *T. molitor*, *B. mori* [34], and *B. mori* [30]). Cruz-Lopez et al. [39] and Ho et al. [35] found similar b* results of sausages with *S. purpurascens* and *A. domesticus*. Choi et al. [28] refers these results to the yellow-colored mealworm. Although b* results of cooked products/sausages with *H. illucens* have not been published so far, the described reasons for the b* results after cooking for these insect species could be also assumed.

Figure 2 shows images of the cooked meat products before and after cooking and of the round slices before packaging in modified atmosphere packages.

**Figure 3:**
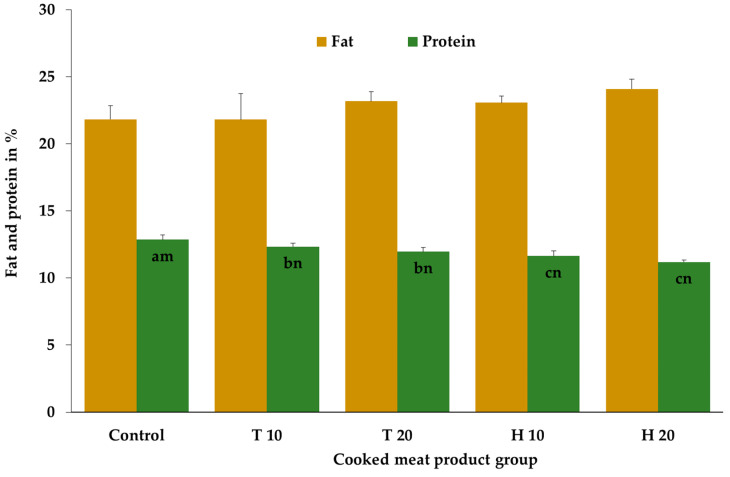
Mean and standard deviation values of the fat and protein contents of the cooked meat products after cooking depending on the meat product group; T 10/T 20 consist of 10%/20% homogenized *Tenebrio molitor* larvae, H 10/H 20 consist of 10%/20% homogenized *Hermetia illucens* larvae; ^abc^ bar graphs with different letters differ significantly (*p* ≤ 0.05) between the species (N = 6 per species) and controls (N = 3); ^mn^ bar graphs with different letters differ significantly (*p* ≤ 0.05) between the concentrations (0% (N = 3), 10% (N = 6), 20% (N = 6)).

The fat contents were comparable between the meat product groups, although the results after higher concentrations of both insects were tendentially higher (*p* = 0.08). The protein content of the control cooked meat products was significantly higher compared to the products with *T. molitor* and *H. illucens*. Additionally, the *Hermetia* products showed significantly lower protein results compared to the cooked meat products with *T. molitor*, independent of the insect concentration added. No significant effect of the insect concentration, independent of the insect species, on the protein concentrations could be obtained (Figure 3).

In contrast to the present study, other publications presented significantly higher fat values in cooked products after the addition of *T. molitor* [28,34], *H. illucens* [29], *B. mori* [30,34] or *A. domesticus* [35]. Other studies also found comparable fat contents using *A. domesticus* [37] or *S. purpurascens* [39]. Similar to the present study, Bessa et al. [29] also found significantly lower protein contents in products with *H. illucens*. However, in many other publications, significantly higher or partly similar protein values in cooked products were found after the addition of *T. molitor* [28,34], *B. mori* [30,34]), *A. domesticus* [35,37] or *S. purpurascens* [39]. The protein and fat results in the present study indicate that the whole larvae of *T. molitor* and *H. illucens* added consist of nearly similar fat but lower protein contents, especially of *Hermetia*, than the pork and beef. The principal discrepancy of the present results compared to other publications might be due to the differing preparation of the insects (drying, boiling) before addition. We used whole larvae, whereas many other authors used dried or otherwise heated insect larvae, which clearly has an impact on the proximate composition results of the insects [6]. Besides this, fat and protein concentration are also influenced by factors such as the species, age, or feeding of the insects [7,10,11,20,40,41,42,43]. Due to this, already published data on the protein and fat composition of *H. illucens* and *T. molitor* are quite variable. Several publications presented only the nutrient composition related to the dry matter, e.g., [8,9,10,11,42,44,45]. Considering different publications that presented the protein and fat content of the whole insects and after recalculation of published data to the whole insect nutrient composition (considering dry matters for *T. molitor* of 37% to 39% and *H. illucens* of 28% to 33%), whole larvae of *T. molitor* consist of 16.6% to 20.5% protein and 8.2% to 14.1% fat [8,9,40,41,42,43,46]. Whole larvae of *H. illucens* consist of 9.1% to 14.4% protein and 10.2% to 12.3% fat [10,11,29,44,45]. These nutrient composition data support the lower protein results in the products, especially with *Hermetia*, as the protein content of pork and beef is 18.0% to 23.6% higher [12,13,14,47,48]. The intramuscular fat (IMF) content of pork and beef is quite variable, ranging, depending on the muscle, from 1.0% to 6.5% [12,13,14,47,48]. For the production of the meat products, pork and beef with an IMF of around 5% and 6%, respectively, were used, which indicates at first that replacing the meat with insects should result in higher fat contents of the cooked meat products. However, the fat content of the cooked meat products is mainly due to the addition of pork fat and to a lesser extent due to the addition of pork, beef, and insects. However, it should also be taken into account that fat analysis has a certain variability.

### 3.2. Analysis of the Cooked Meat Product during Modified Atmosphere Storage

During all storage days, the cooked meat products of the different groups had comparable water activity results with mean values of 0.971 ± 0.002 on day 7 and 0.970 ± 0.002 on day 21. In another publication [39], comparable a_w_ values of sausages with or without grasshopper flour were presented despite significant differences in the moisture results. However, as far as we know, studies with packaged products with insects considering the a_w_ values have not been published.

On day 7 of storage, L* values of the control and *T. molitor* samples were significantly higher compared to the cooked meat products with *Hermetia*, independent of the concentration. Besides this, independent of the insect species, higher percentages (20%) of the insects resulted in lower L* values compared to the 10% and control samples (*p* ≤ 0.05). However, on day 21 the lightness results were comparable between the groups, probably due to the high variation of the L* results on this day of storage (Table 3).

On day 7, the a* values of the cooked meat products during modified atmosphere storage were significantly influenced by the species, concentration, and their interaction, while on day 21, it was only by the insect species. Considering the interaction effects on day 7, the a* values of the control samples were significantly higher than those of the other groups. The results of the T 10, T 20, and H 10 products were comparable (*p* > 0.05), differing from the H 20 and control meat product results. On day 7 of storage, a* values of the control cooked meat products were significantly higher than the results of the *Tenebrio* and *Hermetia* products, which also showed significantly different a* results. With an increased addition of the insects, the a* values decreased significantly (*p* ≤ 0.05), independent of the insect species. On day 21 of storage, products with *Hermetia* had significantly lower a* values compared to the control samples. The a* results of the *Tenebrio* cooked meat products were comparable with those of the other groups (Table 3).

The yellowness (b*) was only significantly influenced by the species, concentration, and their interaction on day 7 of storage. Control samples showed the lowest b* values compared to the products of the other groups. The latter had comparable b* results. Increasing the addition of insects resulted in an increase in the b* values, independent of the insect species. Interestingly, day 7 samples with 20% *Tenebrio* had the highest, and the control samples had the lowest b* values compared to the other groups. The results of the T 10, H 10, and H 20 were comparable (*p* > 0.05), differing significantly from the T 20 and control samples (Table 3).

The storage in a modified atmosphere up to day 7 resulted in nearly similar L* and b* results compared to the meat products after cooking. Interestingly, further storage up to day 21 aligns the lightness and yellowness appearance of the products. The a* values were much more influenced by the storage, especially in the *Hermetia* cooked meat products. This difference was already visible after cooking the products, but the values were not significantly different at this time due to their high variation. In general, a high variation of the color values on day 21 has to be considered, indicating an irregular effect of the modified atmosphere within the packages during longer storage. As the consumer is used to a particular appearance of products, darker, less red, and more yellow products might influence their buying behavior.

Considering the sensory analysis, on day 7 of storage, the cooked meat products with the insects showed significantly lower sensory values after opening of the packages in comparison to the control samples. The products with *Hermetia* also had significantly lower sensory values than the *Tenebrio* products. On day 21, the difference in the sensory results was only significant between the *Hermetia* and the control cooked meat products. With regard to the effect of the concentration, higher concentrations resulted in a significant reduction in the sensory results on day 7, but not on day 21. However, the differences in the sensory values were not only influenced by the appearance but also by the smell of the products (Table 3). The reduced sensory results of the insect products principally agree with their color values, mainly the lower lightness and redness and the higher yellowness results. Different studies support our sensory results [28,39,40,41]. For example, Choi et al. [28] presented that frankfurters with increasing amounts of *T. molitor* showed reduced color, flavor, and overall acceptability results. Cruz-Lopez et al. [39] found reduced overall acceptability results after the addition of grasshopper flour to cooked sausages, Karnjanapratum et al. [49] found reduced overall acceptance of chicken bread spread with *B. mori* and Cavalheiro et al. [50] found reduced appearance, color, and general acceptability after replacing frankfurter meat with flour of *A. domesticus*.

The hardness values of the control cooked meat products were significantly higher on days 7 and 21 of storage than the products with the insects. Additionally, the products with *Tenebrio* had significantly higher hardness results compared to the *Hermetia* cooked meat products on day 21. On day 21, increasing the insect concentration from 10% to 20% decreased the hardness results. The gumminess values were significantly reduced on day 7 by the addition of *H. illucens* and on day 21 by the addition of both insects compared to the control meat products. On day 7, the gumminess values of the cooked meat products with *Tenebrio* were comparable with those of the other products. The cohesiveness and chewiness results of the control meat products are tendentially higher than those of the insect products, but the results are not significantly different (Table 4). The hardness and gumminess results in the present study mainly agree with other studies using *T. molitor* [28,33], *H. illucens* after 14 d storage [29], or *Z. morio* [27]. No effect of the replacement of meat with insects was shown for *H. illucens* after 1 d storage [29] or *A. domesticus* [35], whereas some publications presented increasing hardness results after the addition of *T. molitor* [34], *B. mori* [30,34], or *A. domesticus* [37]. The missing effect on the cohesiveness and chewiness results in the present study seem at first to be due to the unexpectedly high variation in the data. However, for example, Choi et al. [28] or Kim et al. [33] also found, despite clear effects on the hardness and gumminess results, no comparable impact of the addition of *Tenebrio* on the cohesiveness values. The same effect was presented by Bessa et al. [29] with *H. illucens*, Park et al. [30] with *B. mori*, and Kim et al. [37] with *A. domesticus*. Kim et al. [34] stated that the increased hardness is an inevitable result of the higher solid compounds in the product. This assumption is principally supported by other studies [30,35,37]. Considering this, the lower hardness results in the present study could be explained by the reduced solid compounds mainly due to the reduction in the protein concentrations of the cooked meat products. However, Bessa et al. [29] found, in Vienna-style sausages with *Hermetia*, despite higher solid compounds, similar hardness values on day 1 and lower results after 14 d of vacuum storage at 4 °C.

The microbiological analysis of the inoculated cooked meat products resulted in no significant differences between the different groups on days 0, 7, and 21 of storage (Table 5). After cooking, the meat products could be contaminated by bacteria, for example, during slicing and packaging. The source of this contamination might be human handling or the raw material (meat, fat, insects, additives (e.g., salt)). To imitate this, we inoculated the products before packaging with *L. monocytogenes*, *E. coli*, or *B. cereus* and analyzed the colony-forming units at days 0, 7, and 21 of modified atmosphere storage. We wanted to establish whether the addition of insects might influence the behavior (pro- or antimicrobial) of the bacteria added. As far as we know, comparable packaging and storage studies have not been published, but some publications show that insects consist of components with potential antimicrobial properties. For example, Flores et al. [51] analyzed the antimicrobial properties of enzymatic hydrolysates of protein extracts of adult *T. molitor* and found effects of these extracts on some bacteria species (e.g., *Bacillus* spp., *Proteus vulgaris*), but unfortunately, not on *Listeria monocytogenes*. With regard to the fat component, it is long known that, for example, medium-chain fatty acids (MCFA) which are made of 6 to 12 C atoms and their derivatives have antimicrobial properties [52]. MCFAs such as caprylic acid (C8:0) or lauric acid are interesting fatty acids with regard to their antibacterial properties [52,53,54,55]. As lauric acid is an important fatty acid of *H. illucens*, this component was also discussed in the publications of Spranghers et al. [20] with regard to its antimicrobial properties or more in general for insects by Borrelli et al. [55]. Considering the insect component chitin, Islam et al. [56] stated that chitin and its derivatives (chitosan) have strong antimicrobial activities. Whereas the insects consist of, as already stated, 50 to 150 g chitin/kg dry matter, chitosan, the deacetylated and water-soluble derivative of chitin, is usually not present in insects. The molecular weight of chitin seems to influence its antimicrobial properties [56], indicating that degradation of the polysaccharide is necessary to cause bacteria-reducing effects. From the presented microbiological results, it could be suggested that the concentrations of (possible) antimicrobial components from the insects were too low to cause a significant effect on the bacteria number during storage.

## 4. Conclusions

The addition of insect larvae of *T. molitor* and *H. illucens* during the production of cooked meat products influences the physicochemical properties of the products not only directly after production but also during storage in modified atmosphere packages. These alterations are important for food producers and consumers. The higher cooking losses after the addition of 20% insects result in direct economic losses for the food producers. The alterations of the physicochemical and sensory parameters have an indirect impact on the food producers, as consumers have certain expectations regarding meat products. The changes in the color values, accompanied by reduced redness and higher lightness and yellowness values, reduced hardness (softening), lower protein values and worse sensory results of the meat products with insects, especially with *H. illucens*, might reduce the consumer acceptance. However, it is positive that the addition of the insects does not result in the alteration of the bacteria growth after inoculation with different bacteria species. Although in the present study procedures such as freezing at −80 °C for the killing of the larvae or autoclavation for the elimination of bacterial contamination are not applicable on an industrial scale, the physicochemical and sensory results indicate that food producers should critically evaluate the use of insects as a protein alternative to pork, beef, or poultry. The general problem is that consumers mainly take into account products that they already eat when making their decisions. Therefore, in the present study, the physicochemical and sensory results are compared with a “standard product” without insects. Further studies on food quality, food safety, and especially consumer acceptance are urgently needed, as alternative (insect-based) protein sources can supply future consumers with protein. It is an important task for future product development, on the one hand, to improve insect-based protein sources, for example, by using food technological methods, and on the other hand, to (slowly) change consumer expectations to prevent (or mitigate) consumers from comparing new products with the old ones. However, this will be a great challenge.

## Figures and Tables

**Table 1:** Composition of the cooked meat product.

Ingredient/Group	Control	*Tenebrio molitor*		*Hermetia illucens*	
		T 10	T 20	H 10	H 20
Pork ^1^	40	32	26	32	26
Beef ^1^	16	14	10	14	10
Water ^1^	20	20	20	20	20
Pork fat ^1^	24	24	24	24	24
*Tenebrio molitor* ^1^	0	10	20	0	0
*Hermetia illucens* ^1^	0	0	0	10	20
Curing salt NaNO_2_^2^	20	20	20	20	20
Glucose ^2^	5	5	5	5	5
Phosphate ^2^	6	6	6	6	6
Ascorbic acid ^2^	3	3	3	3	3

^1^ Ingredients in %; ^2^ ingredients in g/kg meat homogenate; T 10/T 20 consist of 10%/20% homogenized *Tenebrio molitor* larvae; H 10/H 20 consist of 10%/20% homogenized *Hermetia illucens* larvae.

**Table 2:** Mean and standard deviation (SD) values of the lightness (L*), redness (a*), and yellowness (b*) of the cooked meat products depending on the meat product group.

Meat Product Group	L* Values		a* Values		b* Values	
	Mean	SD	Mean	SD	Mean	SD
Control	70.0 ^am^	0.1	11.4	4.0	15.3 ^by^	1.4
T 10	66.8 ^abn^	0.6	11.5	0.5	17.3 ^ayx^	0.1
T 20	62.7 ^abo^	1.7	10.4	0.4	19.1 ^ax^	0.4
H 10	64.1 ^bn^	1.0	9.8	0.8	17.7 ^ax^	0.7
H 20	57.6 ^bo^	2.2	6.9	0.4	17.5 ^ax^	0.3
*p* value S	0.011		0.076		0.0032	
*p*-value C	0.0001		0.086		0.18	
*p*-value S*C	0.15		0.412		0.048	

T 10/T 20 consist of 10%/20% homogenized *Tenebrio molitor* larvae, H 10/H 20 consist of 10%/20% homogenized *Hermetia illucens* larvae; ^ab^ bar graphs with different letters differ significantly (*p* ≤ 0.05) between the species (N = 6 per species) and controls (N = 3) (S); ^mno^ bar graphs with different letters differ significantly (*p* ≤ 0.05) between the concentrations (C, 0% (N = 3), 10% (N = 6), 20% (N = 6)); ^xy^ bar graphs with different letters differ significantly (*p* ≤ 0.05) between the interaction of species and concentrations (S*C, N = 3).

**Table 3:** Mean and standard deviation (SD) values of the lightness (L*), redness (a*), and yellowness (b*) and the sensory results of the cooked meat products on days 7 and 21 of storage in modified atmosphere (70% N_2_, 30% CO_2_) depending on the meat product group.

Meat Product Group	L* Values				a* Values				b* Values				Sensory Analysis			
	Day 7		Day 21		Day 7		Day 21		Day 7		Day 21		Day 7		Day 21	
	Mean	SD	Mean	SD	Mean	SD	Mean	SD	Mean	SD	Mean	SD	Mean	SD	Mean	SD
Control	70.1 ^am^	0.4	65.9	0.4	13.8 ^amx^	0.2	13.0 ^a^	2.4	14.6 ^boz^	0.3	14.4	2.9	4.8 ^ax^	0.2	3.8 ^a^	1.1
T 10	66.9 ^an^	1.0	71.0	1.0	11.5 ^bny^	0.6	12.5 ^ab^	1.3	17.3 ^any^	0.4	17.2	2.7	3.6 ^by^	0.1	3.2 ^ab^	0.8
T 20	62.9 ^ao^	1.3	68.1	1.3	10.4 ^boy^	0.6	11.1 ^ab^	0.5	19.0 ^amx^	0.4	19.1	1.8	2.8 ^by^	0.3	2.7 ^ab^	0.5
H 10	63.5 ^bn^	0.7	66.2	6.1	9.8 ^cny^	0.4	10.2 ^b^	1.0	17.2 ^any^	1.5	18.9	1.3	2.4 ^cz^	0.5	2.3 ^b^	1.0
H 20	57.3 ^bo^	2.0	62.4	2.0	6.2 ^coz^	1.1	8.6 ^b^	2.0	17.1 ^amy^	0.2	17.5	1.9	1.3 ^cz^	0.3	1.1 ^b^	0.0
*p*-value S	0.0013		0.36		0.0003		0.018		<0.0001		0.85		<0.0001		0.0094	
*p*-value C	<0.0001		0.42		<0.0001		0.13		0.0003		0.96		0.003		0.096	
*p*-value S*C	0.15		0.90		0.0066		0.91		0.001		0.22		0.29		0.45	

T 10/T 20 consist of 10%/20% homogenized *Tenebrio molitor* larvae, H 10/H 20 consist of 10%/20% homogenized *Hermetia illucens* larvae; ^abc^ columns with different letters differ significantly (*p* ≤ 0.05) between the species (N = 6 per species) and controls (N = 3) (S); ^mno^ columns with different letters differ significantly (*p* ≤ 0.05) between the concentrations (C, 0% (N = 3), 10% (N = 6), 20% (N = 6)); ^xyz^ columns with different letters differ significantly (*p* ≤ 0.05) between the interaction of species and concentrations (S*C, N = 3).

**Table 4:** Mean and standard deviation (SD) values of different texture parameters of the cooked meat products on days 7 and 21 of storage in modified atmosphere (70% N_2_, 30% CO_2_) depending on the meat product group.

Meat Product Group	Hardness (N)				Gumminess (N)				Cohesiveness				Chewiness (N)			
	Day 7		Day 21		Day 7		Day 21		Day 7		Day 21		Day 7		Day 21	
	Mean	SD	Mean	SD	Mean	SD	Mean	SD	Mean	SD	Mean	SD	Mean	SD	Mean	SD
Control	27.2 ^a^	5.9	32.3 ^am^	2.2	1624 ^a^	811	2138 ^a^	175	583	911	797	1268	959	1094	1297	1131
T 10	19.5 ^b^	6.7	25.7 ^bn^	1.5	1249 ^ab^	267	1685 ^b^	225	476	711	646	1010	842	807	1016	904
T 20	16.7 ^b^	2.1	19.5 ^bo^	0.7	1047 ^ab^	202	1250 ^b^	133	415	626	477	714	610	549	747	663
H 10	16.8 ^b^	1.4	17.8 ^cn^	4.5	959 ^b^	302	1055 ^c^	577	395	592	191	213	597	584	827	725
H 20	11.3 ^b^	0.6	12.5 ^co^	2.9	633 ^b^	103	717 ^c^	217	224	288	186	213	437	379	515	446
*p* value S	0.0038		0.0001		0.04		0.0008		0.81		0.48		0.64		0.51	
*p*-value C	0.11		0.0039		0.29		0.056		0.76		0.85		0.64		0.55	
*p*-value S*C	0.59		0.76		0.80		0.79		0.88		0.86		0.93		0.96	

T 10/T 20 consist of 10%/20% homogenized *Tenebrio molitor* larvae, H 10/H 20 consist of 10%/20% homogenized *Hermetia illucens* larvae; ^abc^ columns with different letters differ significantly (*p* ≤ 0.05) between the species (N = 6 per species) and controls (N = 3) (S); ^mno^ columns with different letters differ significantly (*p* ≤ 0.05) between the concentrations (C, 0% (N = 3), 10% (N = 6), 20% (N = 6)).

**Table 5:** Mean and standard deviation (SD) values of the numbers of different bacteria species (in log_10_ colony forming units/g meat product), inoculated on cooked meat products, analyzed on days 7 and 21 of storage in a modified atmosphere (70% N_2_, 30% CO_2_) depending on the meat product group.

Meat Product Group	*Bacillus cereus*						*Listeria monocytogenes*						*Escherichia coli*					
	Day 0		Day 7		Day 21		Day 0		Day 7		Day 21		Day 0		Day 7		Day 21	
	Mean	SD	Mean	SD	Mean	SD	Mean	SD	Mean	SD	Mean	SD	Mean	SD	Mean	SD	Mean	SD
Control	4.0	0.3	3.6	0.3	2.7	1.0	6.3	0.4	6.1	0.2	5.9	0.5	6.5	0.3	6.5	0.6	6.4	0.8
T 10	3.9	0.3	2.3	1.3	2.1	1.6	6.3	0.1	6.2	0.2	5.9	0.6	6.3	0.5	6.5	0.5	6.2	0.3
T 20	4.0	0.2	2.4	1.4	1.7	1.8	6.6	0.3	6.1	0.3	5.9	0.8	6.5	0.5	6.4	0.5	6.2	0.2
H 10	4.1	0.6	2.1	0.6	2.1	1.4	6.3	0.1	6.3	0.2	5.9	1.1	6.4	0.5	6.4	0.6	6.2	0.4
H 20	4.0	0.4	2.5	1.1	2.4	1.3	6.2	0.1	6.2	0.2	6.0	0.6	6.6	0.5	6.4	0.5	5.9	0.2
*p* value S	0.61		0.94		0.66		0.19		0.46		0.92		0.73		0.91		0.52	
*p*-value C	0.99		0.70		0.95		0.74		0.73		0.90		0.45		0.83		0.48	
*p*-value S*C	0.58		0.78		0.73		0.14		0.95		0.90		0.96		0.97		0.59	

T 10/T 20 consist of 10%/20% homogenized *Tenebrio molitor* larvae, H 10/H 20 consist of 10%/20% homogenized *Hermetia illucens* larvae; (N = 3).

## Data Availability

Data is contained within the article.
